# Effect of seasonality on the estimated mean value of nutrients and ranking ability of a self-administered diet history questionnaire

**DOI:** 10.1186/1475-2891-13-51

**Published:** 2014-05-31

**Authors:** Hitomi Suga, Keiko Asakura, Satoshi Sasaki, Masanori Nojima, Hitomi Okubo, Naoko Hirota, Akiko Notsu, Mitsuru Fukui, Chigusa Date

**Affiliations:** 1 Department of Social and Preventive Epidemiology, Graduate School of Medicine, The University of Tokyo, Tokyo, Japan; 2 Interfaculty Initiative in Information Studies, Graduate School of Interdisciplinary Information Studies, The University of Tokyo, Tokyo, Japan; 3 Department of Social and Preventive Epidemiology, School of Public Health, The University of Tokyo, Hongo 7-3-1, Bunkyo-ku, Tokyo 113-0033, Japan; 4 Division of Advanced Medicine Promotion, The Advanced Clinical Research Center, The Institute of Medical Science, The University of Tokyo, Tokyo, Japan; 5 Department of Health and Nutritional Science, Faculty of Human Health Science, Matsumoto University, Nagano, Japan; 6 Food Science and Nutrition Department, Tottori College, Tottori, Japan; 7 Department of Statistics, Osaka City University Medical School, Osaka, Japan; 8 Department of Food Science and Nutrition, School of Human Science and Environment, University of Hyogo, Hyogo, Japan

**Keywords:** Diet history questionnaire, Nutrient intake, Seasonality, Validity

## Abstract

**Background:**

We examined the effect of seasonality on the validity (ability to estimate the mean intake of a group and ranking ability) of nutrient intakes estimated with a comprehensive self-administered diet history questionnaire (DHQ) developed for the assessment of Japanese diets during the preceding one month, using semi-weighed dietary records (DRs) as a reference method.

**Methods:**

This study was conducted in three areas in Japan (Osaka, Nagano, and Tottori). The study population included 92 Japanese men aged 32–76 years and 92 Japanese women aged 31–69 years (30 from Osaka, 31 from Nagano, and 31 from Tottori for each sex). A DHQ and a four-day DR were completed four times at 3-month intervals, once per season. The effect of seasonality was examined by the level of agreement among seasons using mean nutrient intake and correlation coefficients.

**Results:**

Significant differences in estimated energy-adjusted intakes of 42 selected nutrients between the average of DRs administered 16 times throughout a year and that of the DHQ administered four times in each season (fall, winter, spring, and summer) were observed for 30, 29, 30, and 31 nutrients for men and 21, 28, 30, and 31 nutrients for women, respectively. Pearson correlation coefficients between the DRs and the DHQs for energy-adjusted intakes of the 42 nutrients showed significant inter-season differences in 11 nutrients for men and 13 nutrients for women. Particularly, correlation coefficients of fat, monounsaturated fat, polyunsaturated fat, n-6 polyunsaturated fat, α-linolenic acid, and cholesterol in spring and cryptoxanthin in summer for men, and fat, saturated fat, and monounsaturated fat in spring and summer and thiamin and iron in summer for women were markedly altered by seasonality.

**Conclusions:**

Mean nutrient intake estimated by the DHQ varied by season, indicating that any consideration of nutrient intake estimated by the DHQ as a yearly average intake may be problematic. In contrast, the effect of seasonality on the ranking ability of the DHQ was relatively small, and thus the use of a DHQ to rank individuals by nutrient intake is acceptable for epidemiological studies, regardless of season.

## Background

Epidemiologic studies in the nutritional field strongly depend on the validity of the methods used to estimate the habitual nutrient intake and food consumption of participants. Dietary questionnaires place a low burden on the respondent, take relatively little time to complete, and are cost effective in large populations, and are often used to estimate dietary intake [1]. Dietary questionnaires are commonly administered a single time only, and nutrient intake estimated from the questionnaire is regarded as the yearly average intake.

As food and nutrient intake vary by season [2-6], many questionnaires attempt to avoid the effects of seasonality by asking about the intake of food during the previous year. However, individual responses with respect to past diet are strongly influenced by the most recent diet [7-9], and because data obtained from dietary questionnaires usually reflect recent rather than ‘habitual’ intake, they can be affected by the season of administration. If nutrient intake data estimated in a particular season are markedly different from habitual nutrient intake, the observed association between habitual dietary exposure and disease could be biased [6,10]. In addition, the season itself should be considered unsuitable for implementation of epidemiological research.

The influence of seasonality on the validity of a dietary questionnaire can be addressed from several aspects. The most common methods are comparison of the means and standard deviations, which assess absolute levels of nutrient intake [1], and the correlation coefficients, which assess the ability to rank subjects according to their nutrient intake [1]. To avoid spurious estimation, however, the validity of dietary questionnaires would be better assessed when the errors of the questionnaire are made as independent as possible from the reference method. In other words, assessing the real impact of seasonality on a questionnaire requires a reference method whose error source is independent from that of the questionnaire, for example dietary records. However, of the three previous studies which reported the effect of seasonality on the nutrient intake estimated by dietary questionnaire [11-13], the average nutrient intake values estimated by the questionnaires replicated multiple times were used as reference. This similarity of error source may cause the effect of seasonality to be underestimated.

Sasaki et al. have developed a comprehensive self-administered diet history questionnaire (DHQ) for the assessment of Japanese diets. The DHQ is a self-administered, semi-quantitative questionnaire that asks about the consumption frequency and portion size of selected foods to estimate dietary intake during the preceding month [14]. The validation study of the DHQ reported that it has satisfactory ranking ability for the energy-adjusted intakes of many nutrients, although its ability to estimate mean values was satisfactory for only a limited number of nutrients [15]. Because it assesses dietary intake in the previous month, the DHQ appears to be vulnerable to seasonal variability in nutrient intake. Nevertheless, the effects of seasonality on its ability to estimate mean values and to rank individuals by nutrient intake have not been examined.

Here, we investigated the effect of seasonality on the validity of the DHQ from the perspective of absolute level and ranking ability. We also used the DHQ to evaluate whether there are seasons which are unsuitable for estimating habitual nutrient intake, using a 16-day dietary record (DR) as reference.

## Methods

### Participants

Details of the study design, participant recruitment strategy, and dietary assessment methods have been reported elsewhere [15,16]. Briefly, the study was conducted in three areas of Japan, Osaka (urban), Nagano (rural inland), and Tottori (rural coastal). In each area, we recruited apparently healthy women aged 30 to 69 years who were willing to participate and who lived together with their husbands, such that each 10-year age class (30–39, 40–49, 50–59, and 60–69 years) contained eight women equally without consideration to the age of their husbands. Thus, a total of 96 women and 96 men were invited. Information sessions for the subjects were held before the study at which the study purpose and protocol were explained. The study did not undergo ethical approval because it was conducted before ethical guidelines for epidemiologic research were enforced in Japan. However, it was conducted according to the guidelines laid down in the Declaration of Helsinki, and use of data from the study was approved by the Research Ethics Committee of the University of Tokyo Faculty of Medicine (No. 3421). Written informed consent was obtained from all subjects.

Finally, 92 men (30 from Osaka, 31 from Nagano, and 31 from Tottori) aged 32 to 76 years and 92 women (30 from Osaka, 31 from Nagano, and 31 from Tottori) aged 31 to 69 years were included in the final analysis sample.

### Dietary records

Participants completed a 4-nonconsecutive-day semi-weighed DR four times, once in each season, at intervals of approximately three months: DR1 in November or December 2002 (autumn), DR2 in February 2003 (winter), DR3 in May 2003 (spring), and DR4 in August or September 2003 (summer). Each set of four recording days consisted of one weekend day and three weekdays.

During the orientation session, registered dietitians gave the participants written and verbal instructions on how to maintain the DR, recording sheets, and a digital scale, and asked them to record and weigh all foods and beverages consumed on each recording day. All collected records were checked by trained dietitians at the respective local center and then again at the study center. A total of 1290 food and beverage items appeared in the DRs. Intakes of energy and 42 selected nutrients were estimated based on the Standard Tables of Food Composition in Japan, 2010 [17].

### Dietary questionnaire

The participants were also required to answer the DHQ four times, once in each season: DHQ1 in November or December 2002 (autumn), DHQ2 in February 2003 (winter), DHQ3 in May 2003 (spring), and DHQ4 in August or September 2003 (summer). The DHQ was administered approximately 2 days before the start of each dietary recording period.

Details of the DHQ have been described elsewhere [15,16]. Briefly, the DHQ is a 16-page structured self-administered questionnaire that asks about the consumption frequency and portion size of selected foods to estimate the dietary intake of 150 food and beverage items during the preceding month. The DHQ consists of seven sections: (1) general dietary behavior; (2) usual cooking methods; (3) consumption frequency and amount of alcoholic beverages; (4) consumption frequency and semi-quantitative portion size of selected food and non-alcoholic beverage items; (5) dietary supplements; (6) consumption frequency and semi-quantitative portion size of staple foods (rice, other grains, noodles, bread and other grain products), soup for noodles, and miso (fermented soybean paste) soup, with questions on the size of cups (bowls) usually used for rice and miso soup; and (7) open-ended items for foods consumed regularly (≥ once/week) but not appearing in the DHQ. The food and beverage items were selected as foods commonly consumed in Japan. Standard portion sizes and sizes of bowls for rice and cups for miso soup were derived from several recipe books for Japanese dishes. Estimates of dietary intake for a total of 150 food and beverage items were calculated using a computer algorithm developed specifically for the DHQ. Information on dietary supplements (section (5)) and data from the open-ended questionnaire items (section (7)) were not used in the calculation of dietary intake because few participants reported the use of dietary supplements or consumption of open-ended items.

### Statistical analysis

We examined the validity of energy and nutrient intake values derived from the DHQ administered in each season from the perspective of absolute level and ranking ability using mean nutrient intake values and correlation coefficients, respectively. We used the average value derived from the 16-day DR administered in four seasons (four-day DR in each season) as reference.

Intakes of energy and 42 selected nutrients were estimated on the basis of the intakes of food items obtained using the DR, or with the DHQ and the Standard Tables of Food Composition in Japan, 2010 [17]. As nutrient intakes were not normally distributed, logarithmic transformation was carried out before analysis. Values of energy intake are shown as crude values. Since most nutrient intakes were positively correlated with energy intake, we used energy-adjusted values by the density method for analysis.

Mean dietary intake and interquartile range were calculated for each assessment method. Statistically significant differences between the DR and DHQ were determined by a paired t-test using two-sided values. P values less than 0.05 were considered to indicate significant differences. We also calculated Pearson correlation coefficient between the DR and the each of the DHQs. Statistically significant differences of correlation coefficients between fall and the other seasons were examined using the Meng-Rosenthal-Rubin method, which is used to compare overlapping correlation coefficients [18]. The tested correlation coefficient pairs are considered statistically different when z is greater than 1.96, with a significance level of 5%. When there were significant differences in mean values or correlation coefficients between fall and the other seasons, we considered that the influence of seasonality existed. All statistical analyses were conducted using the SAS statistical software package version 9.2 (SAS Institute Inc., Cary, NC, USA) for women and men separately.

## Results

Basic characteristics of the participants are shown in Table 1. The seasonal means of energy and nutrient intakes calculated from the DR and DHQ are shown in Table 2 for men and Table 3 for women. Energy intakes estimated by the DHQ were significantly lower than those by DR, except for winter for men, and fall and winter for women. Regarding the intake of 42 nutrients, significant differences between the DR and DHQ administered in fall, winter, spring, and summer were observed for 30, 29, 30, and 31 nutrients for men and 21, 28, 30, and 31 nutrients for women, respectively.

**Table 1:** Basic characteristics of the study population (mean values and standard deviations (SD))

		**Men (n = 92)**		**Women (n = 92)**
	**Mean**	**SD**	**Mean**	**SD**
Age, years	52.8	12.1	49.6	11.4
Body height, cm	168.0	6.7	155.6	5.8
Body weight, kg	66.2	11.2	53.4	7.1
Body mass index, kg/m^2^	23.3	3.1	22.1	2.6

**Table 2:** Comparison of mean crude energy intakes and energy-adjusted nutrient intakes estimated by the 16-day DR, and the DHQ administered in each season among 92 men

	**Men (n = 92)**					
	**DR**	**IQR**	Fall^*^	Winter^*^	Spring^*^	Summer^*^
Energy, kJ/day	9804	8709-10756	9300*	9768	8085***	8889**
Protein, % energy	14.2	13.2-15.2	12.9***	12.9***	13.1***	12.7***
Fat, % energy	24.6	22.5-27.7	23.2*	23.7	26.6*	25.5
Saturated fat, % energy	6.68	5.88-7.62	5.97***	6.15***	7.04	6.93
Monounsaturated fat, % energy	8.72	7.69-10.2	8.28*	8.47	9.89***	9.36**
Polyunsaturated fat, % energy	5.68	5.10-6.36	5.61	5.64	6.34**	6.02*
n-3 Polyunsaturated fat, % energy	1.13	0.96-1.28	1.11	1.10	1.29**	1.25**
n-6 Polyunsaturated fat, % energy	4.52	3.98-5.14	4.52	4.57	5.09***	4.81*
Marine-origin n-3 polyunsaturated fat^†^, % energy	0.44	0.32-0.55	0.36***	0.35***	0.41	0.42
Eicosapentaenoic acid, % energy	0.15	0.11-0.19	0.13**	0.12***	0.14	0.14
Docosahexaenoic acid, % energy	0.25	0.18-0.31	0.21***	0.20***	0.23	0.24
α-Linolenic acid, % energy	0.62	0.54-0.71	0.70***	0.70**	0.82***	0.77***
Cholesterol, mg/10 MJ	400	349-464	361**	362**	422	417
Carbohydrate, % energy	53.7	50.8-57.8	54.4	53.0	43.0***	42.4***
Total dietary fiber, g/10 MJ	15.5	12.8-18.7	13.7***	13.2***	11.3***	11.2***
Soluble dietary fiber, g/10 MJ	3.35	2.74-4.05	3.18	3.11**	2.75***	2.89***
Insoluble dietary fiber, g/10 MJ	11.4	9.39-13.8	10.0***	9.62***	8.03***	7.89***
Alcohol, % energy	3.40	0.82-9.13	3.75	3.87	7.38***	9.63***
Retinol, μg/10 MJ	293	179-341	241	254	345	351
Vitamin A	633	460-777	521**	511**	646	645
(retinol equivalent)^‡^, μg/10 MJ						
α-Carotene, μg/10 MJ	418	320-583	221***	202***	237***	210***
β-Carotene, μg/10 MJ	3104	2490-4170	2291***	2066***	2657*	2639**
β-Carotene equivalent^§^, μg/10 MJ	3522	2884-4665	2659***	2381***	2924**	2857***
Cryptoxanthin, μg/10 MJ	232	130-458	317**	249	139***	97.4***
α-Tocopherol, mg/10 MJ	8.37	7.51-9.32	8.23	8.28	9.53***	9.46***
Vitamin K, μg/10 MJ	241	185-313	250	241	279***	236
Thiamin, mg/10 MJ	1.09	1.00-1.18	0.95***	0.94***	0.96***	0.94***
Riboflavin, mg/10 MJ	1.51	1.32-1.70	1.45	1.41**	1.92***	1.88***
Niacin, mg/10 MJ	21.4	19.4-23.4	19.7**	19.5***	28.1***	30.0***
Vitamin B6, mg/10 MJ	1.49	1.34-1.64	1.37***	1.32***	1.86***	1.97***
Vitamin B12, μg/10 MJ	9.25	6.82-12.3	8.66	8.83	10.6**	10.7**
Folate, μg/10 MJ	392	327-479	319***	309***	412	404
Pantothenic acid, mg/10 MJ	7.12	6.39-7.71	6.79**	6.66***	7.46**	7.49***
Vitamin C, mg/10 MJ	112	88.1-154	108	92.9***	105	101*
Sodium, mg/10 MJ	5000	4571-5592	4750*	4621**	4630**	4451***
Potassium, mg/10 MJ	2937	2635-3290	2582***	2448***	3019	3138***
Calcium, mg/10 MJ	585	493-733	503***	495***	601	572
Magnesium, mg/10 MJ	315	281-365	288***	287***	356***	365***
Phosphorus, mg/10 MJ	1267	1149-1380	1159***	1154***	1331**	1323**
Iron, mg/10 MJ	9.25	8.10-10.5	7.74***	7.64***	7.76***	7.34***
Zinc, mg/10 MJ	9.94	9.15-10.8	9.44**	9.51**	8.39***	7.97***
Copper, mg/10 MJ	1.42	1.26-1.54	1.31***	1.30***	1.08***	1.06***
Manganese, mg/10 MJ	4.27	3.63-5.07	4.58*	4.23	3.96	3.76**

Abbreviations: DR = semi-weighed 16-day dietary records; DHQ = self-administered diet history questionnaire; IQR = interquartile range.

All variables were log-transformed before analysis. Means were back-transformed.

^*^The periods in which the DHQs were administered in each season were as follows: Fall = November or December 2002, Winter = February 2003, Spring = May 2003, Summer = August or September 2003.

^†^Sum of eicosapentaenoic acid, docosapentaenoic acid, and docosahexaenoic acid.

^‡^Sum of retinol, β-carotene/12, α-carotene/24, and cryptoxanthin/24.

^§^Sum of β-carotene, α-carotene/2, and cryptoxanthin/2.

Significant difference from the DR: *P < 0.05, **P < 0.01, ***P < 0.001 (paired t-test).

**Table 3:** Comparison of mean crude energy intakes and energy-adjusted nutrient intakes estimated by the 16-day DR, and the DHQ administered in each season among 92 women

	**Women (n = 92)**					
	**DR**	**IQR**	Fall^*^	Winter^*^	Spring^*^	Summer^*^
Energy, kJ/day	7722	7071-8589	7854	7682	6621***	7028**
Protein, % energy	15.1	14.0-16.4	14.0***	13.9***	14.4*	13.9***
Fat, % energy	27.3	25.0-30.6	27.5	26.7	30.3***	29.0*
Saturated fat, % energy	7.71	6.68-8.84	7.43	7.37*	8.50***	8.30*
Monounsaturated fat, % energy	9.50	8.33-11.0	9.52	9.23	10.8***	10.3**
Polyunsaturated fat, % energy	6.23	5.76-6.70	6.42	6.22	7.03***	6.69*
n-3 Polyunsaturated fat, % energy	1.20	1.04-1.37	1.26	1.18	1.41***	1.35***
n-6 Polyunsaturated fat, % energy	5.00	4.56-5.55	5.17	5.06	5.66***	5.36*
Marine-origin n-3 polyunsaturated fat^†^, % energy	0.44	0.32-0.55	0.38**	0.35***	0.44	0.43
Eicosapentaenoic acid, % energy	0.15	0.11-0.18	0.13	0.12***	0.15	0.15
Docosahexaenoic acid, % energy	0.25	0.19-0.31	0.22**	0.20***	0.25	0.24
α-Linolenic acid, % energy	0.70	0.62-0.77	0.82***	0.79***	0.90***	0.87***
Cholesterol, mg/10 MJ	432	374-497	386**	370***	448	430
Carbohydrate, % energy	55.1	51.7-58.7	55.5	56.3*	46.1***	46.0***
Total dietary fiber, g/10 MJ	18.8	16.1-22.3	17.6**	17.0***	15.2***	14.7***
Soluble dietary fiber, g/10 MJ	4.15	3.44-4.83	4.20	4.06	3.67***	3.81**
Insoluble dietary fiber, g/10 MJ	13.7	11.8-16.0	12.7***	12.3***	10.7***	10.2***
Alcohol, % energy	0.78	0.14-1.62	0.62*	0.49***	2.53***	2.90***
Retinol, μg/10 MJ	331	224-423	307	282*	392*	440***
Vitamin A	741	574-857	717	662*	814	844*
(retinol equivalent)^‡^, μg/10 MJ						
α-Carotene, μg/10 MJ	480	340-661	437	425	386**	353***
β-Carotene, μg/10 MJ	3764	2763-5368	3583	3439	3833	3781
β-Carotene equivalent^§^, μg/10 MJ	4330	3159-6029	4173	3957	4251	4118
Cryptoxanthin, μg/10 MJ	355	225-661	506***	374	223***	147***
α-Tocopherol, mg/10 MJ	9.57	8.66-10.5	9.79	9.61	11.1***	11.0***
Vitamin K, μg/10 MJ	295	244-362	330**	335**	369***	302
Thiamin, mg/10 MJ	1.17	1.06-1.26	1.05***	1.03***	1.08*	1.09*
Riboflavin, mg/10 MJ	1.71	1.50-1.91	1.73	1.63*	2.08***	2.09***
Niacin, mg/10 MJ	21.4	19.2-24.0	20.2**	19.2***	25.3***	25.7***
Vitamin B6, mg/10 MJ	1.57	1.34-1.77	1.45***	1.38***	1.79***	1.84***
Vitamin B12, μg/10 MJ	9.55	7.47-12.1	9.03	8.92	10.8**	10.5*
Folate, μg/10 MJ	452	368-552	392***	377***	455	427*
Pantothenic acid, mg/10 MJ	7.78	7.16-8.39	7.76	7.57	8.33***	8.39***
Vitamin C, mg/10 MJ	139	119-185	148	122***	134	131
Sodium, mg/10 MJ	5320	4767-6027	5364	5043*	5120	5096
Potassium, mg/10 MJ	3408	2918-3849	3146***	2954***	3411	3544*
Calcium, mg/10 MJ	729	615-876	701	677**	787**	760
Magnesium, mg/10 MJ	350	306-394	325***	321***	372***	372***
Phosphorus, mg/10 MJ	1383	1263-1521	1315**	1291***	1450**	1438*
Iron, mg/10 MJ	10.5	9.15-12.1	9.05***	8.89***	9.12***	8.58***
Zinc, mg/10 MJ	10.4	9.80-11.2	10.0***	10.3	9.41***	9.15***
Copper, mg/10 MJ	1.52	1.37-1.68	1.44***	1.47*	1.28***	1.26***
Manganese, mg/10 MJ	4.61	3.89-5.59	5.17***	4.85*	4.63	4.46

Abbreviations: DR = semi-weighed 16-day dietary records; DHQ = self-administered diet history questionnaire; IQR = interquartile range.

All variables were log-transformed before analysis. Means were back-transformed.

^*^The periods in which the DHQs were administered in each season were as follows: Fall = November or December 2002, Winter = February 2003, Spring = May 2003, Summer = August or September 2003.

^†^Sum of eicosapentaenoic acid, docosapentaenoic acid, and docosahexaenoic acid.

^‡^Sum of retinol, β-carotene/12, α-carotene/24, and cryptoxanthin/24.

^§^Sum of β-carotene, α-carotene/2, and cryptoxanthin/2.

Significant difference from the DR: *P < 0.05, **P < 0.01, ***P < 0.001 (paired t-test).

Pearson correlation coefficients for estimates of nutrient intake and energy intake on the DR versus the DHQ administered in each season are shown in Table 4 for men and Table 5 for women. The correlation values of energy with the DR versus DHQ administered in fall, winter, spring, and summer ranged from 0.31 (spring) to 0.40 (fall and winter) for men, and from 0.19 (spring) to 0.30 (fall) for women, respectively. There were no significant differences between fall and the other seasons; that is, there may be no effect of seasonality on the ability of the DHQ to rank subject according to energy intake. Regarding the 42 nutrients, the medians (interquartile ranges) of correlation coefficients on administration in fall, winter, spring, and summer were 0.47 (0.36-0.56), 0.48 (0.37-0.58), 0.40 (0.28-0.53), and 0.41 (0.33-0.51) for men, and 0.50 (0.43-0.59), 0.48 (0.40-0.59), 0.43 (0.34-0.52), and 0.40 (0.32-0.50) for women, respectively. The number of nutrients which showed significant differences was largest in spring for men (8 nutrients) and in summer for women (10 nutrients) and lowest in winter for men and women (1 nutrient in each). Significant differences in correlation coefficients between fall and at least one of other season were shown for 11 nutrients for men and 13 nutrients for women. Among them, most values were significantly lower than those in fall, except for zinc in winter and vitamin K in spring for men, and calcium in spring and manganese in winter for women. This implies that the ranking ability of these nutrients may vary by season. Particularly, correlation coefficients of fat, monounsaturated fat, polyunsaturated fat, n-6 polyunsaturated fat, α-linolenic acid, and cholesterol in spring and cryptoxanthin in summer for men, and fat, saturated fat, and monounsaturated fat in spring and summer and thiamin and iron in summer for women were markedly altered by seasonality.

**Table 4:** Pearson correlation coefficients between the 16-day DR and DHQ administered in each season for estimated crude energy and energy-adjusted nutrient intake among 92 men

	**Men (n = 92)**			
	Fall^*^	Winter^*^	Spring^*^	Summer^*^
Energy	0.40	0.40	0.31	0.36
Protein	0.34	0.47	0.46	0.40
Fat	0.53	0.59	0.27*	0.43
Saturated fat	0.56	0.68	0.40	0.47
Monounsaturated fat	0.58	0.61	0.25**	0.44
Polyunsaturated fat	0.48	0.43	0.21**	0.33
n-3 Polyunsaturated fat	0.32	0.17	0.19	0.28
n-6 Polyunsaturated fat	0.50	0.49	0.24**	0.35
Marine-origin n-3 polyunsaturated fat^†^	0.36	0.34	0.40	0.36
Eicosapentaenoic acid	0.36	0.34	0.41	0.38
Docosahexaenoic acid	0.36	0.33	0.37	0.33
α-Linolenic acid	0.39	0.37	0.11*	0.30
Cholesterol	0.46	0.55	0.19*	0.41
Carbohydrate	0.64	0.63	0.52	0.58
Total dietary fiber	0.72	0.73	0.69	0.60
Soluble dietary fiber	0.64	0.66	0.64	0.60
Insoluble dietary fiber	0.72	0.73	0.68	0.59*
Alcohol	0.82	0.87	0.65***	0.66**
Retinol	0.19	0.32	0.23	0.18
Vitamin A(retinol equivalent)^‡^	0.20	0.30	0.25	0.25
α-Carotene	0.10	0.24	0.18	0.30
β-Carotene	0.36	0.47	0.49	0.52
β-Carotene equivalent^§^	0.38	0.48	0.46	0.51
Cryptoxanthin	0.54	0.46	0.35	0.21**
α-Tocopherol	0.42	0.37	0.29	0.27
Vitamin K	0.46	0.52	0.67**	0.54
Thiamin	0.36	0.31	0.20	0.21
Riboflavin	0.38	0.39	0.49	0.48
Niacin	0.43	0.45	0.40	0.44
Vitamin B6	0.54	0.51	0.35	0.40
Vitamin B12	0.38	0.38	0.32	0.36
Folate	0.35	0.46	0.39	0.27
Pantothenic acid	0.57	0.64	0.58	0.49
Vitamin C	0.49	0.52	0.44	0.47
Sodium	0.33	0.36	0.32	0.32
Potassium	0.53	0.56	0.62	0.57
Calcium	0.66	0.67	0.72	0.69
Magnesium	0.57	0.48	0.56	0.51
Phosphorus	0.53	0.51	0.59	0.55
Iron	0.49	0.56	0.50	0.37
Zinc	0.33	0.51*	0.40	0.35
Copper	0.59	0.63	0.46	0.46
Manganese	0.49	0.48	0.53	0.40

Abbreviations: DR = semi-weighed 16-day dietary records; DHQ = self-administered diet history questionnaire.

All variables were log-transformed before analysis.

^*^The periods in which the DHQs were administered in each season were as follows: Fall = November or December 2002, Winter = February 2003, Spring = May 2003, Summer = August or September 2003.

^†^Sum of eicosapentaenoic acid, docosapentaenoic acid, and docosahexaenoic acid.

^‡^Sum of retinol, β-carotene/12, α-carotene/24, and cryptoxanthin/24.

^§^Sum of β-carotene, α-carotene/2, and cryptoxanthin/2.

Significant difference between correlation coefficients fall and other seasons: *P < 0.05, **P < 0.01, ***P < 0.001(Meng-Rosenthal-Rubin method).

**Table 5:** Pearson correlation coefficients between 16-day DR and DHQ administered in each season for estimated crude energy and energy-adjusted nutrient intake among 92 women

	**Women (n = 92)**			
	Fall^*^	Winter^*^	Spring^*^	Summer^*^
Energy	0.30	0.26	0.19	0.28
Protein	0.45	0.37	0.40	0.28
Fat	0.59	0.62	0.30**	0.36*
Saturated fat	0.70	0.68	0.47**	0.46**
Monounsaturated fat	0.55	0.64	0.26**	0.33*
Polyunsaturated fat	0.35	0.41	0.22	0.29
n-3 Polyunsaturated fat	0.23	0.22	0.24	0.19
n-6 Polyunsaturated fat	0.44	0.50	0.27	0.35
Marine-origin n-3 polyunsaturated fat^†^	0.39	0.43	0.41	0.31
Eicosapentaenoic acid	0.42	0.45	0.47	0.39
Docosahexaenoic acid	0.36	0.40	0.36	0.27
α-Linolenic acid	0.23	0.29	0.22	0.23
Cholesterol	0.35	0.30	0.34	0.33
Carbohydrate	0.62	0.63	0.43*	0.54
Total dietary fiber	0.69	0.66	0.61	0.60
Soluble dietary fiber	0.62	0.55	0.52	0.54
Insoluble dietary fiber	0.70	0.69	0.62	0.59
Alcohol	0.85	0.89	0.79	0.68***
Retinol	0.43	0.40	0.31	0.41
Vitamin A (retinol equivalent)^‡^	0.47	0.35	0.34	0.35
α-carotene	0.47	0.31	0.34	0.40
β-Carotene	0.58	0.43	0.55	0.53
β-Carotene equivalent^§^	0.58	0.47	0.57	0.54
Cryptoxanthin	0.47	0.56	0.36	0.33
α-Tocopherol	0.39	0.42	0.40	0.29
Vitamin K	0.47	0.42	0.59	0.47
Thiamin	0.31	0.35	0.14	0.02*
Riboflavin	0.50	0.44	0.42	0.27*
Niacin	0.52	0.39	0.39	0.31*
Vitamin B6	0.60	0.58	0.44	0.42*
Vitamin B12	0.46	0.33	0.33	0.26
Folate	0.54	0.49	0.50	0.42
Pantothenic acid	0.57	0.60	0.60	0.51
Vitamin C	0.51	0.55	0.50	0.41
Sodium	0.38	0.35	0.30	0.36
Potassium	0.49	0.51	0.52	0.49
Calcium	0.50	0.56	0.67**	0.54
Magnesium	0.60	0.59	0.51	0.52
Phosphorus	0.44	0.45	0.52	0.45
Iron	0.63	0.50	0.48	0.36**
Zinc	0.50	0.54	0.42	0.35
Copper	0.67	0.66	0.57	0.50*
Manganese	0.47	0.64*	0.59	0.47

Abbreviations: DR = semi-weighed 16-day dietary records; DHQ = self-administered diet history questionnaire.

All variables were log-transformed before analysis.

^*^The periods in which the DHQs were administered in each season were as follows: Fall = November or December 2002, Winter = February 2003, Spring = May 2003, Summer = August or September 2003.

^†^Sum of eicosapentaenoic acid, docosapentaenoic acid, and docosahexaenoic acid.

^‡^Sum of retinol, β-carotene/12, α-carotene/24, and cryptoxanthin/24.

^§^Sum of β-carotene, α-carotene/2, and cryptoxanthin/2.

Significant difference between correlation coefficients fall and other seasons: *P < 0.05, **P < 0.01, ***P < 0.001(Meng-Rosenthal-Rubin method).

## Discussion

We examined the effect of seasonality on the ability of the DHQ to estimate the mean nutrient intake of a population and to rank individuals according to nutrient intake, using DRs collected over a total of 16 days as reference. Mean nutrient intake estimated by the DHQ varied according to season, indicating that any consideration of nutrient intake estimated by the DHQ as a yearly average intake may be problematic. The DHQ is a questionnaire which assesses the previous month’s dietary intake, and seasonal variance in mean values estimated by it may therefore represent the true seasonal variance of nutrient intake. On the other hand, the correlation coefficients between the DR and each of the questionnaires showed significant differences for some nutrients, suggesting that seasonality affected the ranking ability of the DHQ for a limited number of nutrients.

Several previous studies in Japan reported that the mean intake of some nutrients varied by season [2-4]. Specifically, Tokudome et al. observed remarkable seasonal differences for carotenes, vitamin A, vitamin C, iron, zinc, arachidonic acid, n-3 polyunsaturated fat, eicosapentaenoic acid, docosahexaenoic acid , soluble dietary fiber, insoluble dietary fiber, and total dietary fiber [4]. Mori et al. reported significant seasonal differences for protein, fat, calcium, iron, vitamin A, thiamine, riboflavin, niacin, and vitamin C [3]. Also, Owaki et al. reported significant seasonal differences for water, vegetable protein, vegetable fat, potassium, vitamin C, vitamin E, sodium, monounsaturated fat, polyunsaturated fat, α-linolenic acid, insoluble dietary fiber, total dietary fiber, and magnesium in men and women, fat and iron in men, and soluble dietary fiber in women [2]. These results imply that the seasonality of nutrition intake can affect mean nutrient intake estimated by dietary questionnaires in epidemiological studies. However, few studies have investigated how the season of dietary questionnaire administration influences the ranking ability of dietary questionnaires. In the present study, we examined seasonal influence on both the estimated mean values and ranking ability of a dietary questionnaire using 16-day dietary records conducted in each season as the reference. To our knowledge, this is the first study to examine the impact of seasonality on the validity (the ability to estimate mean intake and ranking ability) of a dietary questionnaire using dietary records as reference.

In this study, the mean intake for energy and most of the 42 nutrients estimated by the DHQ differed significantly from those estimated by the DR, and varied according to season. Kobayashi et al. reported significant differences between mean intakes estimated by the DHQ and by the DR for energy and many energy-adjusted nutrients [15]. Our present results are consistent with these findings as well as those of other studies of the effect of seasonality [11-13]. This result implies that regarding the mean value of a nutrient’s intake estimated by the DHQ as the yearly average intake is problematic. However, the DHQ assesses dietary intake for the previous month, and the seasonal variance in mean values estimated by it might therefore represent the true seasonal variance of nutrient intake.

We also found a significant difference in correlation coefficients between fall and the other seasons in 11 nutrients for men and 13 for women. This implies that seasonality can affect the ability to rank individuals according to their intake of these nutrients. Most of the significant differences in correlation coefficients were shown in spring and summer for both sexes. It is not clear why most of the significant differences in correlation coefficients were observed in spring and summer. We examined how many food and beverage items appeared in the DR of the participants in each season and how many food and beverage items were used in the DHQ’s computer algorithm, which also appeared in the DR (data not shown). There was no significant seasonal difference in the number of food and beverage items appeared in the DR and the DHQ. Thus the seasonal difference observed in this study might have been mainly caused by the difference of intake amount in foods and beverages contained in the DHQ. However, we also found the number of vegetables, fish, and shellfishes included in the DHQ’s computer algorithm was considerably smaller compared with that appeared in the DR. Since intake of vegetables and fish tended to be affected by season, it is possible that the number of vegetables and fish included in the DHQ’s nutritional value calculation is not enough to reflect the seasonal change of intake for these foods. Our results indicate that there may be no particular season which is unsuitable for ranking the individual’s habitual intake of nutrients by dietary questionnaire, except for a limited number of nutrients. For example, summer might not be suitable for dietary assessment in studies examining the relationship between thiamin intake and health outcomes for women, because the correlation coefficient of thiamine for women in summer was extremely low (0.02).

These results imply the need for caution in administering epidemiological studies which extend over two or more seasons. Although seasonal effects on the correlation coefficients between seasons were relatively small, absolute nutrient intake varies according to season. Thus, the combination and analysis of data collected in different seasons can be problematic. If the purpose of using a questionnaire is to rank individuals according to nutrient intake, we recommend that the questionnaire be administered in a single season.

We acknowledge several limitations of this study. First, we may have not discriminated between “seasonal change in nutrient intake” and the possible influence of repetition of the dietary questionnaire. Repeated experience with dietary questionnaires and dietary records might change participants’ attention to food consumption and improve the precision of assessment by DHQ 2–4. On this basis, subjects’ attention to food consumption might be misinterpreted as ‘seasonal difference’. However, the limitations of dietary questionnaires largely relate to the simplification of dietary intake into a modest number of multiple choice questions, rather than to subject recognition and recall of food [19]. Moreover, our present study found no significant improvement in correlation coefficient between DHQ1 and DHQ 2–4, except for zinc and vitamin K for men, and calcium and manganese for women. We therefore consider that the effect of repetition, if present, is not large. Second, the DHQ asks about the consumption frequency and portion size of selected foods during the preceding month and is likely more vulnerable to seasonality. Consequently, the results of this study are applicable to similar questionnaires that ask about food intake for a preceding month, and might not be generalizable to questionnaires that ask about the yearly intake of foods. This means that the effect of seasonality observed in our study is likely larger than that seen in other studies using other dietary questionnaires. Third, although we used mean values of energy and nutrient intake derived from the 16-day DR as reference, DRs are susceptible to day-to-day variability in nutrient intake. A DR collection period of 16 days and our sample size (92 men and 92 women) might therefore be too short and small for consideration as usual individual intake, particularly for micronutrients. Finally, our subjects were volunteers, and may therefore have been more nutritionally conscious than others who did not participate in the study. Accordingly, they might not be representative of the general Japanese population, and our results might thus not be generalizable to the Japanese population.

## Conclusions

This study showed that the DHQ has acceptable ranking ability over timescales of 1 year, although the ability to estimate mean values is reasonable for only a limited number of nutrients regardless of the season of dietary questionnaire administration. Our findings suggest that values for energy and most nutrients estimated on single administration of the DHQ are acceptable for large-scale epidemiological studies in Japan.

## Abbreviations

DHQ: Self-administered diet history questionnaire; DR: Semi-weighed dietary record; SD: Standard deviation.

## Competing interests

The authors declare that they have no competing interests.

## Authors’ contributions

HS designed the study, analyzed the data, and wrote the manuscript. KA assisted in writing and editing the manuscript. SS organized the study, and contributed to data collection, writing, and editing the manuscript. MN assisted data analysis. HO, NH, AN, MF, and CD were involved in study design, data collection and data management. All authors read and approved the final manuscript.
